# Anti‐PF4 disorders: Pathogenesis, diagnosis and treatment

**DOI:** 10.1111/bjh.20216

**Published:** 2025-07-01

**Authors:** Megan V. Preece, Devi V. Pathak, Mike Laffan, Deepa J. Arachchillage

**Affiliations:** ^1^ Centre for Haematology, Department of Immunology and Inflammation Imperial College London London UK; ^2^ Department of Haematology Imperial College Healthcare NHS Trust London UK

**Keywords:** heparin, heparin‐induced thrombocytopenia, immunoglobulin G, platelet factor 4, platelet‐activating antibodies, thrombosis, vaccine‐induced immune thrombocytopenia and thrombosis

## Abstract

Platelet factor 4 (PF4) is a cationic protein, able to form complexes with negatively charged molecules upon its self‐assembly into PF4 tetramers. The targeting of these PF4 complexes by immunoglobulin G (IgG) antibodies underlies anti‐PF4 disorders such as heparin‐induced thrombocytopenia (HIT) and Vaccine‐Induced Immune Thrombocytopenia and Thrombosis (VITT)/VITT‐like disorders. The formation of IgG/PF4 immune complexes facilitates uncontrolled activation of platelets, neutrophils and monocytes, via IgG‐mediated Fcγ receptor binding. This promotes the thrombocytopenia and thrombosis characteristic of anti‐PF4 disorders. HIT is predominantly triggered by heparin exposure. VITT is a recently recognised anti‐PF4 disorder, which developed following specific SARS‐CoV‐2 vaccinations. It is thought that hexon proteins, components of adenoviral vectors, may form complexes with PF4 to trigger anti‐PF4 antibody production in VITT. A novel anti‐PF4 disorder has been recognised causing platelet activation without the administration of heparin or SARS‐CoV‐2 vaccination and referred to as ‘VITT‐like disorder.’ Clinical evaluation of HIT and VITT/VITT‐like disorders is based on thrombotic events, platelet counts and D‐dimer levels. Laboratory assays such as heparin/PF4‐induced platelet activation assays can be used to distinguish between HIT and VITT. Treatment plans for HIT and VITT may differ across patient groups. In this review, we discuss the pathogenesis, diagnosis and management of anti‐PF4 disorders.

## Graphical abstract

Anti‐platelet factor 4 (PF4) disorders include heparin‐induced thrombocytopenia (HIT) and vaccine‐induced immune thrombocytopenia and thrombosis (VITT)/VITT‐like disorders. The formation of immunoglobulin G (IgG)/PF4 immune complexes facilitates uncontrolled activation of platelets, neutrophils and monocytes via IgG‐mediated Fcγ receptor binding. This promotes the thrombosis and thrombocytopenia characteristic of anti‐PF4 disorders. Laboratory assays such as heparin/PF4‐induced platelet activation assays can be used to distinguish between HIT and VITT. Treatment plans for HIT and VITT may differ across patient groups but predominantly involve stopping heparin, starting non‐heparin anticoagulant for HIT and intravenous Immunoglobulin ± plasma exchange with non‐heparin anticoagulation for thrombosis (VITT)/VITT‐like disorders.

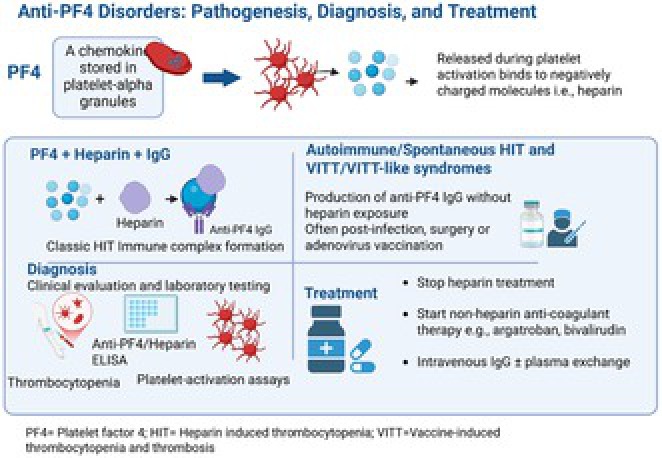

## INTRODUCTION

**FIGURE 1:**
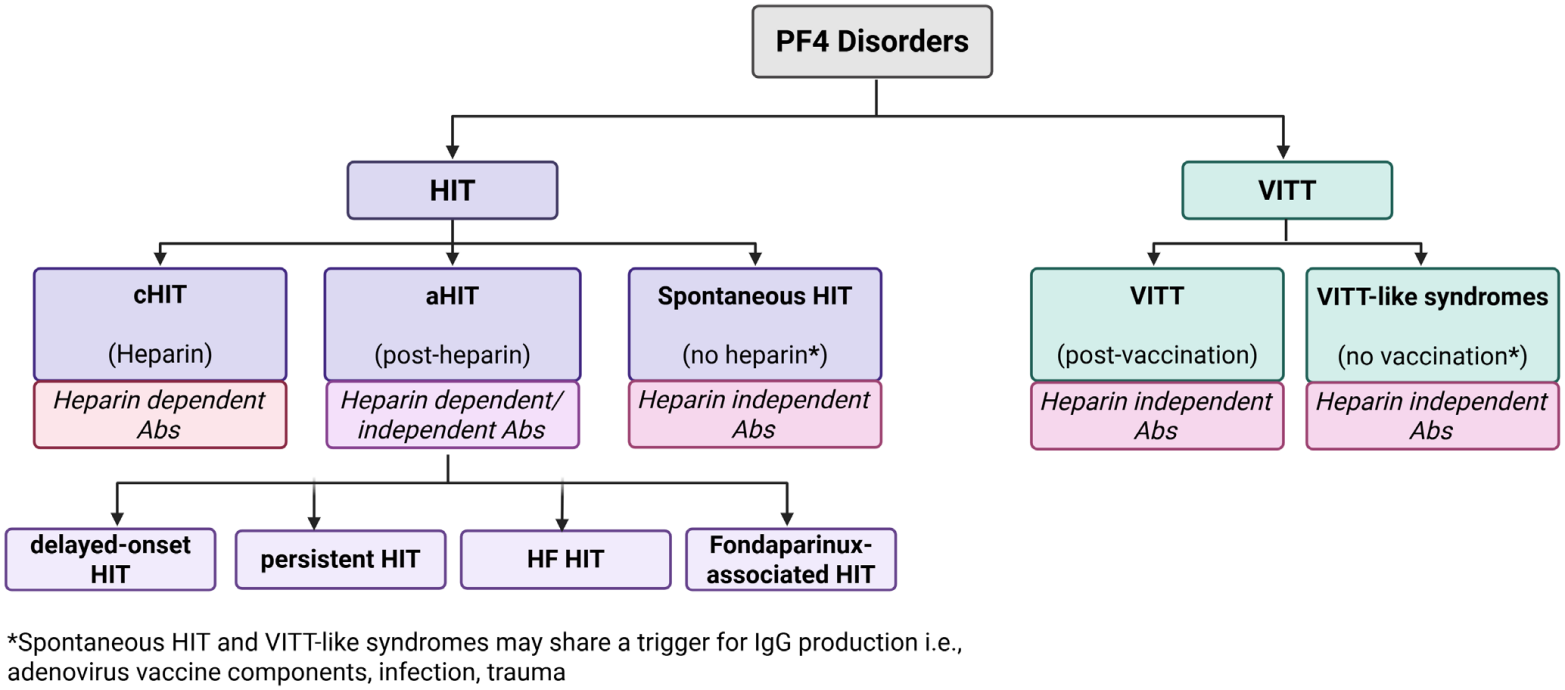
Flow diagram classifying HIT, VITT and VITT‐like syndromes by anti‐PF4 antibodies and heparin dependence. cHIT occurs with heparin exposure and involves the production of heparin‐dependent (anti‐PF4/H) antibodies. In aHIT, which can occur with or without proximate exposure, both heparin‐dependent and heparin‐independent antibodies may be produced. aHIT can be classified into four subtypes: delayed‐onset HIT, persistent HIT, HF HIT and fondaparinux‐associated HIT. Spontaneous HIT exclusively involves the production of heparin‐independent antibodies, occurring in the absence of heparin. VITT, involving heparin‐independent antibodies, has been shown to arise post‐vaccination (predominantly associated with the SARS‐CoV‐2 vaccination programmes). Heparin‐independent antibody production, despite no heparin exposure or vaccination, but could be following adenovirus/other viral infection is termed as a VITT‐like syndrome. Abs, antibodies; aHIT, autoimmune heparin‐induced thrombocytopenia; cHIT, classic heparin‐induced thrombocytopenia; HF, heparin ‘flush’; HIT, heparin‐induced thrombocytopenia; VITT, vaccine‐induced thrombocytopenia and thrombosis.

Platelet factor 4 (PF4) disorders represent a range of immune‐mediated thrombogenic conditions which include heparin‐induced thrombocytopenia (HIT), comprising classic HIT (cHIT), autoimmune HIT (aHIT), delayed onset HIT, persisting (refractory) HIT, heparin ‘flush’ HIT and spontaneous HIT and vaccine‐induced immune thrombocytopenia and thrombosis (VITT)/VITT‐like syndromes. These disorders are characterised by the binding of immunoglobulin G (IgG) antibodies to PF4 complexes, leading to platelet activation and consequent thrombocytopenia and thrombosis (Figure 1). HIT was first recognised as a prothrombotic disorder associated with heparin‐dependent, platelet‐activating antibodies in 1973.^1^ In 1992, the antigenic target for HIT was recognised as cationic chemokine PF4 (also named CXCL4).^2^ Research into these disorders has been propelled by the emergence of VITT cases following SARS‐CoV‐2 vaccination in 2021. In this review, we discuss the pathogenesis, diagnosis and management of anti‐PF4 disorders.

## SEARCH STRATEGY AND SELECTION CRITERIA

We identified references for this review through searches of PubMed and Medline with the search terms ‘platelet factor 4 AND/OR platelet factor 4 disorders’, ‘thrombosis’, ‘platelet‐activating antibodies AND/OR platelet factor 4 antibodies’, ‘Heparin (adverse effects)’ AND platelets ‘Heparin induced thrombocytopenia’ AND/OR vaccine‐induced immune thrombocytopenia and thrombosis like syndrome with ‘diagnosis AND/OR treatment and ‘clinical presentation’ AND/OR spontaneous heparin induced thrombocytopenia’, ‘Diagnosis of HIT (screening tests, immunological assay [ELISA, ACUSTAR] functional assay)’, ‘vaccine‐induced immune thrombocytopenia and thrombosis’, ‘vaccine‐induced immune thrombocytopenia and thrombosis like syndrome’, ‘management of heparin induced thrombocytopenia and/or vaccine‐induced immune thrombocytopenia and thrombosis’, ‘alternative anticoagulants for heparin induced thrombocytopenia’, ‘IV Ig and/or plasma exchange in heparin induced thrombocytopenia or vaccine‐induced immune thrombocytopenia and thrombosis’ or ‘immunoglobulin G’ from 1973 to March 2025. Only peer‐reviewed papers (original and review articles) and abstracts published in English were reviewed. The final reference list was generated on the basis of originality and relevance to the broad scope of this review.

## THE PHYSIOLOGICAL ROLE OF PLATELET FACTOR 4 (PF4)

The *PF4* gene on chromosome 4 encodes a 7.8‐kDa cationic protein abundant in platelet alpha granules. This 70‐amino‐acid monomer assembles into homotetramers, exhibiting chemotactic activity for neutrophils, fibroblasts and monocytes.^3^ PF4 is crucial in innate immunity by binding and opsonizing polyanionic pathogen surfaces.^3^, ^4^

Also known as CXCL4, PF4 is secreted upon platelet activation, forming a complex with platelet chondroitin sulphate proteoglycans (CSPGs) and translocating to endothelial heparan sulphates.^5^ This inhibits antithrombin (AT) activation, promoting coagulation.^6^, ^7^ Unlike other chemokines in the CXC chemokine family, PF4 does not bind to CXCR2, but its highly positively charged state allows it to interact with various binding partners via electrostatic interactions; a vital characteristic in the role of PF4 in the pathogenesis of HIT and VITT/VITT‐like disorders.^8^

## PLATELET‐ACTIVATING ANTIBODIES AGAINST PF4

**FIGURE 2:**
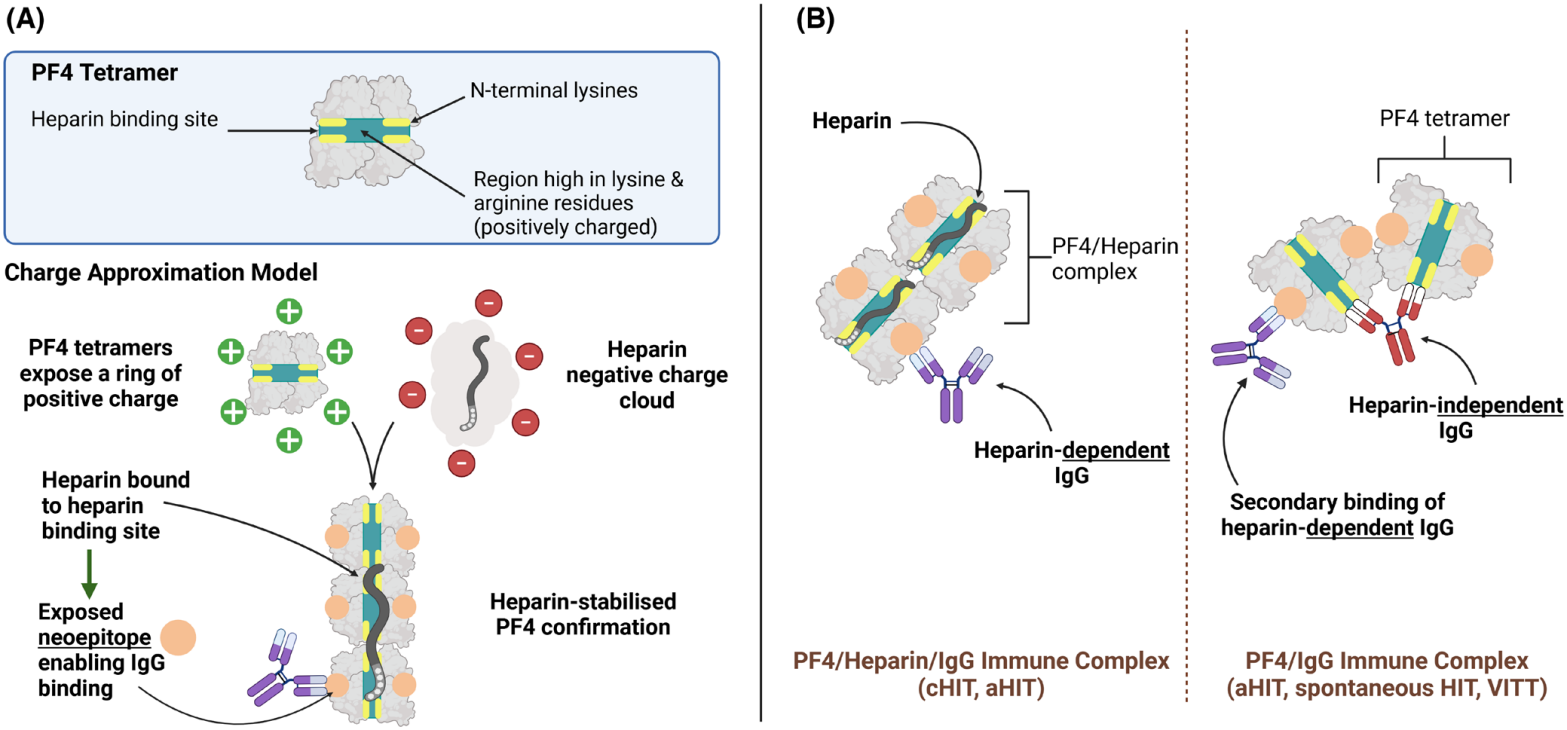
Formation of PF4/IgG immune complexes in HIT and PF4's charge approximation model. Panel (A) PF4 tetramer structure and charge approximation process. PF4 tetramers lose their intermolecular distance as negatively charged polyanions bind to the positively charged PF4 tetramer, neutralising its charge and inducing conformational changes. This exposes neoepitopes, leading to the formation of anti‐PF4/polyanion antibodies, a key step in the pathogenesis of heparin‐induced thrombocytopenia (HIT). This enables the formation of PF4/heparin/IgG complexes leading to platelet activation and thrombosis. Panel (B) PF4 immune complex forms with heparin‐dependent and/or heparin‐independent IgGs, depending on HIT subtype. Heparin‐dependent IgG binds to antigen binding sites on the exterior of the tetramer. HIT‐independent IgG binds to N‐terminal lysines in the heparin binding antigen site. Adapted from [6]. aHIT, autoimmune heparin‐induced thrombocytopenia; cHIT, classic heparin‐induced thrombocytopenia; IgG, immunoglobulin G; PF4, platelet factor 4; VITT, vaccine‐induced thrombocytopenia and thrombosis.

PF4 tetramer formation exposes a ‘ring of positive charge’ formed by the lysine and arginine‐rich PF4 N‐termini (Figure 2A,B) which allows PF4 to complex with negatively charged molecules/polyanions via electrostatic interactions.^3^, ^6^ In HIT, anti‐PF4 IgG antibodies recognise an epitope formed by a heparin‐PF4 complex (here referred to as anti‐PF4/H antibodies, i.e. the antibodies are ‘heparin‐dependent’).^4^, ^6^ The antibody–antigen complex then causes platelet activation by binding to platelet FcγRIIA receptors, promoting a pro‐thrombotic state, that is, further release of PF4, platelet aggregation, release of procoagulant microparticles,^7^ as well as calpain‐dependent thrombocytopenia.^9^, ^10^ Following activation, platelets in HIT can undergo death through complex apoptotic and calpain‐dependent non‐apoptotic pathways.^11^ However, removal of platelets in HIT is mainly by macrophages and neutrophils as discussed in the section on pathogenesis below (see Pathogenesis of thrombosis and thrombocytopenia in anti‐PF4 disorders).

Heparin binding neutralises the PF4 strong positive charge, enabling PF4 tetramers to come into close proximity—a crucial step in forming multimolecular PF4/heparin complexes that triggers an immune response (Figure 2).^3^, ^5^, ^12^ This also allows the formation of large PF4–IgG complexes mediating pancellular activation of platelets, monocytes and neutrophils.^13^, ^14^

Since the recognition of HIT in the 1970s, several HIT subtypes including cHIT, aHIT and spontaneous HIT have been recognised (Figure 1), (see HIT). The classification of these subtypes has led to the recognition that anionic ‘triggering agents’ other than heparin can promote the immune response and that not all antibodies require the trigger to bind PF4 and mediate platelet activation.^15^ Other triggers include pharmaceuticals such as fondaparinux^16^ and pentosan polysulphate^17^, ^18^ as well as postulated bacterial and viral antigens and endogenous sugars (Table 1). The subtypes of HIT are associated with different clinical manifestations, including onset time, clinical associations, degree of thrombocytopenia and varying risk of thrombotic events (see HIT).

**TABLE 1:** HIT triggering polyanions: Their origins and associated antibodies.

Polyanion	Origin	Associated antibodies
Unfractionated heparin (UFH)	Pharmaceutical (sourced from porcine mucosa)	Anti‐PF4 Heparin IgG
Low molecular weight heparin (LMWH)	Pharmaceutical (sourced from porcine mucosa)	Anti‐PF4/Heparin IgG
Chondroitin sulphate	Endogenous (connective tissue matrix)	Anti‐PF4/Chondroitin Sulphate IgG (rare)
Dextran sulphate	Pharmaceutical	Anti‐PF4/Dextran Sulphate IgG
Nucleic acids (DNA & RNA)	Endogenous or viral (released during cell death or infection)	Anti‐PF4/Nucleic Acid IgG
Lipid A	Bacterial surfaces	Anti‐PF4/Lipid A IgG

*Note*: Examples of various polyanionic molecules that interact with PF4, their origins (pharmaceutical, endogenous, synthetic or bacterial) and the associated antibodies formed in response (e.g. anti‐PF4/Heparin IgG).^14^

**FIGURE 3:**
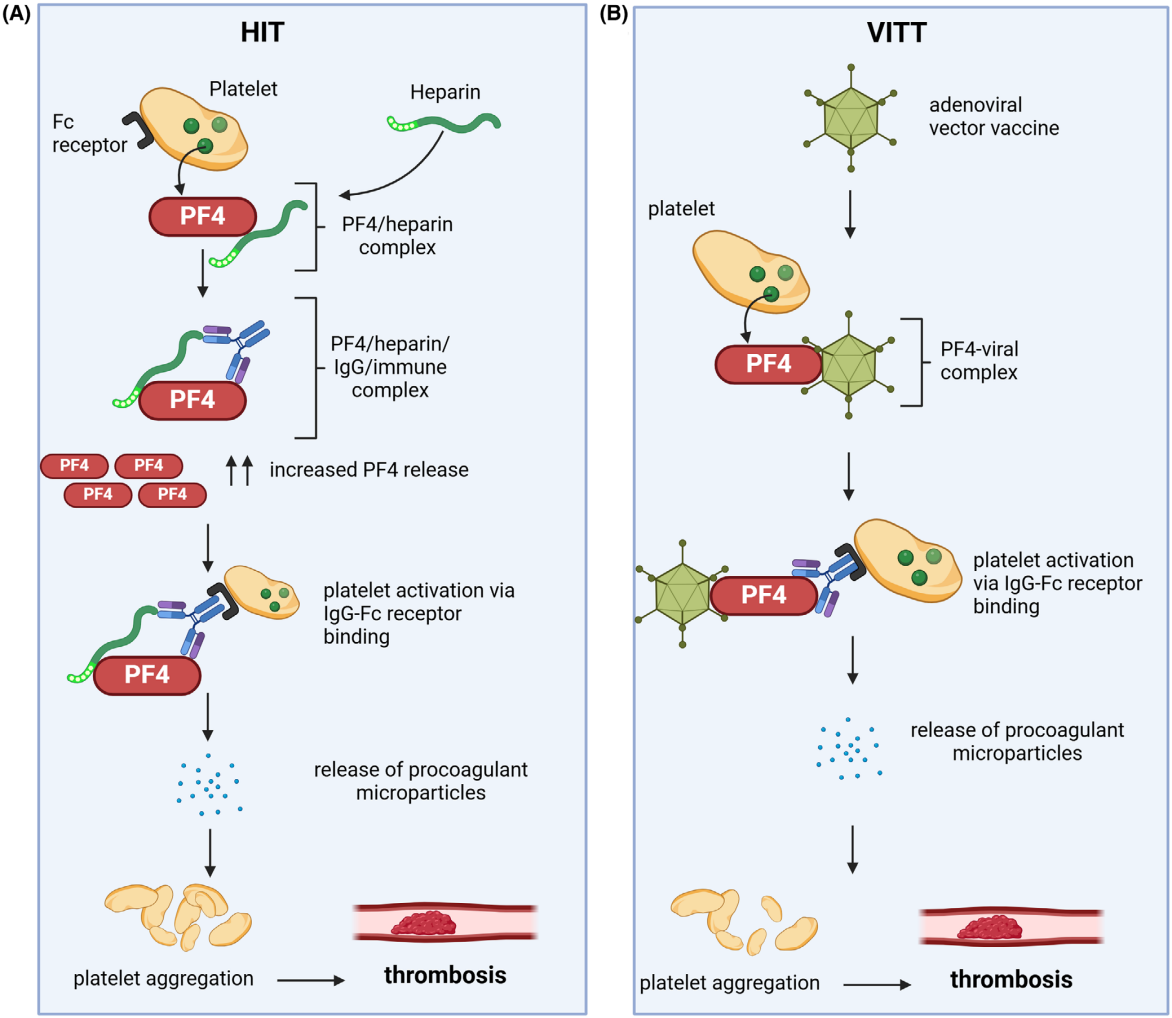
IgG‐FcγRIIa receptor binding is essential for the pathogenesis of thrombosis in HIT and VITT anti‐PF4 disorders. Panel (A) Platelet factor 4 (PF4), released from alpha granules of activated platelets, binds to heparin, forming PF4–heparin complexes. IgG antibodies recognise and bind these complexes, forming PF4–heparin–IgG multimolecular immune complexes. These immune complexes engage platelet FcγRIIa receptors, triggering platelet activation, further PF4 release and amplification of the prothrombotic cascade. This process leads to the release of procoagulant microparticles, platelet aggregation and potential thrombosis. Panel (B) PF4 binds to the adenoviral vector, forming PF4–viral complexes. IgG antibodies recognise these complexes, leading to platelet activation through FcγRIIa receptor engagement. This activation promotes the release of procoagulant microparticles, platelet aggregation and creates a prothrombotic state.^3^, ^4^, ^6^, ^7^ FcγRIIa receptor, Fc gamma receptor IIa; IgG, immunoglobulin G; PF4, platelet factor 4.

Moreover, the antibodies in different anti‐PF4 disorders^19^ recognise different epitopes on PF4. For example, in cHIT, heparin binds to what is referred to as the ‘heparin‐binding site’ on PF4 tetramers (Figure 2A) inducing a conformational change in PF4 and the formation of two heparin‐dependent antigen sites on opposing ends of PF4 tetramers. Anti‐PF4/heparin IgG antibodies bind to this new site, forming platelet‐activating PF4/heparin/IgG immune complexes^10^, ^20^, ^21^ (Figure 3). The identification of a single pathogenic epitope on PF4/Heparin complexes has proven difficult due to the polyclonal nature of HIT antibodies. Nonetheless, PF4 amino acids Cys10,42, Cys12, Thr15, Cys36, Thr38, Ile42, Arg49, Ile51, Cys52 and Asp54 have been identified as important for the binding of both pathogenic and enzyme‐immunoassay (EIA)‐positive/serotonin‐release assay (SRA)‐negative antibodies.^20^ The lower frequency of HIT with LMWH is attributed to shorter chain length and lower sulphation^22^ reducing the ability to form large PF4 complexes.

In the recently recognised anti‐PF4 disorder, VITT, the causative antibodies recognise the heparin‐binding site on PF4 tetramers in the absence of heparin.^18^ Research so far indicates that the production of VITT antibodies is triggered by the binding of adenoviral proteins to PF4 after the administration of adenovirus vector vaccines for SARS‐CoV‐2 such as ChAdOx1 nCov‐19 (Oxford/AstraZeneca).^23^, ^24^ Therefore, VITT antibodies are referred to as being ‘heparin‐independent’. It is also noted that HIT antibodies are polyclonal, while VITT antibodies are monoclonal or oligoclonal.^25^, ^26^ Interestingly, although these are distinct subsets of antibodies, certain anti‐PF4 disorders, notably auto‐immune heparin‐induced thrombocytopenia (aHIT), involve both heparin‐dependent and heparin‐independent antibodies (see Autoimmune HIT (aHIT)).^27^ Furthermore, it seems that antibodies in aHIT do not require the presence of the triggering anion for their PF4 binding.

## HIT

HIT typically develops 5–10 days after heparin administration. However, variations in onset can occur, notably immediately after heparin exposure or several days after discontinuation as observed in ‘delayed HIT’.^5^, ^27^, ^28^

As depicted in Figure 1, HIT can be broadly classified into classic HIT, autoimmune HIT (aHIT) which all occur with proximate heparin exposure, and spontaneous HIT‐like syndromes that develop without proximate heparin exposure. The reported incidence of cHIT ranges from 1 in 5000 to 3–7 in 100 inpatients depending on the patient population, with a higher incidence in patients receiving unfractionated heparin, undergoing major surgery (particularly orthopaedic and cardiac surgery) and patients supported by extracorporeal membrane oxygenation (ECMO).^29^, ^30^, ^31^ The reported incidence of VITT in the United Kingdom is approximately 1:100 000 among patients 50 years of age or older and at least 1:50 000 among patients in the younger group^32^, ^33^ while exact incidences of aHIT and spontaneous HIT and VITT‐like disorders are not known. These disorders are very rare but prompt recognition and treatment are vital to survival of the patients.

### Classic heparin‐induced thrombocytopenia (cHIT)

Classic heparin‐induced thrombocytopenia (cHIT) occurs 5–10 days following heparin administration. IgG antibodies against PF4/Heparin are formed with subsequent immune complex formation. These immune complexes activate platelets via FcγRIIa binding, resulting in thrombocytopenia and platelet activation (see Pathogenesis of thrombosis and thrombocytopenia in anti‐PF4 disorders). Approximately 25%–50% of HIT cases develop thrombotic complications, both venous and arterial.^28^, ^34^ HIT is more common with post‐surgical states, high‐dose heparin, unfractionated heparin and trauma.^35^ Antibodies in cHIT are triggered by heparin exposure, that is, are always heparin‐dependent in comparison to autoimmune HIT, which can be heparin‐dependent or independent.^36^, ^37^

### Autoimmune HIT (aHIT)

Autoimmune heparin‐induced thrombocytopenia (aHIT) includes several distinct subtypes of HIT, characterised by pathological heparin‐dependent and ‐independent IgG‐mediated platelet activation.^14^

#### Delayed onset HIT

A 2001 study by Warkentin and Kelton defined the term ‘delayed onset HIT’ as characterised by a decrease in platelet count occurring at least 5 days after heparin discontinuation, with manifestations extending up to 3 weeks post‐therapy.^38^ Although this follows heparin exposure, the condition is attributed to a heparin‐independent platelet‐activating mechanism, as heparin, with its short half‐life of approximately 60 minutes, would typically be cleared from the patient by this time.

Serological studies conducted on 12 patients with delayed onset HIT, which began an average of 9.2 days following heparin cessation, revealed consistently higher titres of IgG specific to PF4/heparin than in healthy controls. Additionally, they showed significantly higher heparin‐independent and heparin‐dependent serotonin release relative to control HIT sera.^6^, ^39^

Heparin‐independent antibodies often target distinct epitopes on PF4, particularly in the heparin‐binding region (Figure 2), but without requiring heparin for immune complex formation. These epitopes on PF4 tetramers may become exposed due to factors such as endogenous glycosaminoglycans, even after heparin has been cleared from the system. The heparin‐independent nature of these antibodies leads to persistent platelet activation, contributing to delayed and more severe thrombotic events. Delayed onset HIT is less likely to be self‐limiting than cHIT, with platelet counts often taking longer to recover and necessitating prolonged or more intensive anticoagulation therapy.^14^

#### Persisting (refractory) HIT

Persisting or ‘refractory HIT’ is characterised by a delayed platelet count recovery, defined as exceeding 1 week after discontinuing heparin, compared to typical 3–4 days with approximately 90% of patients recovering within 7 days in cHIT,^28^ and an increased risk of thrombosis.

The delayed recovery may be attributed to the persistence of high‐titre pathogenic antibodies, targeting multiple PF4 epitopes and which continue to form platelet‐activating complexes despite the removal of heparin.

Persisting HIT can last several weeks, requiring extended use of non‐heparin anticoagulants. The persistence of platelet‐activating antibodies often correlates with delayed clearance of the immune complexes and continued activation of FcγRIIa receptors on platelets.^40^

#### Heparin ‘flush’ HIT (HF‐HIT)

Heparin flushes comprise low doses of heparin solution (typically ≤200 IU) administered to prevent thrombus formation in catheters and IV devices. Heparin flush HIT (HF‐HIT) is therefore characterised by thrombocytopenia and thrombotic complications despite minimal systemic heparin exposure.^28^

This phenomenon highlights the complex stoichiometry of heparin‐PF4 interactions, where optimal complex formation occurs at specific molar ratios associated with charge neutralisation, resulting in a ‘bell‐shaped’ curve of reactivity that influences antibody formation and clinical manifestations of HIT.^41^

Recognition of HF‐HIT underscores the need for careful monitoring of platelet counts in at‐risk patients and judicious use of heparin flushes, favouring non‐heparin alternatives such as Danaparoid when feasible.^42^ A comprehensive review of the literature suggested that the patency of arterial central lines is feasible with normal saline for several days.^43^

#### Fondaparinux‐induced HIT

The pentasaccharide fondaparinux (composed of five saccharide units) is an indirect factor Xa inhibitor, proposed as a treatment for suspected HIT due to its negligible risk of inducing the condition. It is a synthetic molecule which is not derived from heparin. Nonetheless, there are rare case reports of HIT associated with fondaparinux use, particularly following knee replacement surgery.^14^ It is thought that heparin with at least 12 saccharide units is required to form antigenic complexes with PF4 but in vitro studies confirm that fondaparinux can interact with PF4. One study described two cases of HIT occurring following knee replacement surgery where fondaparinux was administered.^44^ However, it is not possible to say with certainty whether it is the knee replacements themselves rather than the fondaparinux that causes HIT in the cases. Furthermore, the platelet activation and pathogenesis of HIT occur in the absence of heparin exposure and are associated with heparin independent antibodies. Hence, fondaparinux HIT is categorised as aHIT.

Despite this, fondaparinux has been deemed a safe alternative non‐heparin anticoagulant following observational studies.^44^, ^45^

### Spontaneous HIT‐like syndromes

Spontaneous HIT‐like syndromes are considered HIT‐mimicking disorders that arise in the absence of prior exposure to heparin or polyanionic medications.^14^ These syndromes exhibit strong serum‐induced platelet activation at 0 IU/mL heparin, with suppression at heparin concentrations of 100 IU/mL.^46^ The time course for spontaneous HIT‐like syndromes typically involves a rapid onset of thrombocytopenia and thrombosis without prior heparin exposure, followed by persistent PF4 antibody‐mediated platelet activation for several weeks, with antibody levels usually declining within 2–4 weeks, coinciding with platelet count recovery, although the exact duration can vary between patients and may be influenced by treatment.^14^ In spontaneous HIT, pathogenic antibodies can recognise PF4 complexed with non‐heparin polyanions such as lipopolysaccharide (LPS) and glycosaminoglycans or PF4 alone, as observed in VITT and VITT‐like disorders. This would explain the broader range of binding mechanisms and proposed immunologic triggers in these syndromes.^14^

Two distinct subtypes of this condition have been identified: a post‐orthopaedic subtype, primarily following knee arthroplasty, and a medical subtype, typically post‐infection.^4^ Clinically, these conditions are associated with unusual thrombotic events including cerebral venous thrombosis and splanchnic vein thrombosis.

A potential mechanism for these subtypes is exposure of endogenous polyanionic molecules, such as glycosaminoglycans or DNA released during tissue injury or infection, which bind to PF4 and form immunogenic complexes. In the post‐orthopaedic subtype, surgical trauma and subsequent tissue remodelling may lead to local release of such polyanions. The post‐infectious subtype may result from the release of bacterial components, such as lipopolysaccharides, or host‐derived danger‐associated molecular patterns (DAMPs) during infection, which similarly interact with PF4 to initiate an immune response. These mechanisms could explain the heparin‐independent platelet activation and the propensity for thrombotic events in these subtypes.^47^, ^48^ However, it is not clear whether continuous presence of the trigger is required in patients with spontaneous HIT to cause platelet activation and thrombosis.

Proposed features that point to the diagnosis of spontaneous HIT include unexplained thrombocytopenia, thrombosis and lack of previous heparin exposure. The presence of platelet‐activating antibodies will confirm the diagnosis (see Diagnosis of HIT).^14^, ^46^ These proposed criteria aim to prevent overdiagnosis of the syndrome.

## VITT AND VITT‐LIKE DISORDERS

By January 2021, in response to the SARS‐CoV‐2 pandemic, The European Medical Agency had approved two adenoviral (AV)‐based vaccines which encoded the spike glycoprotein of SARS‐CoV‐2. The Oxford‐AstraZeneca SARS‐Cov‐2 vaccine used a recombinant chimpanzee adenovirus vector, ChAdOx1, while the Johnson and Johnson/Janssen vaccine used a recombinant human adenovirus type 26 vector.^23^ Concern was raised when a small number of individuals developed an unusual combination of severe thrombotic events such as splanchnic vein thrombosis and cerebral venous sinus thrombosis (CVST) associated with thrombocytopenia 5–30 days following vaccination.^23^, ^49^

This led to the recognition of VITT, an anti‐PF4 disorder with a pathophysiology closely resembling that of HIT. VITT more closely resembles aHIT than classic HIT because there is no preceding heparin exposure, and the platelet‐activating anti‐PF4 IgG antibodies bind in the absence of heparin.^23^, ^24^ Using biophysical techniques and mouse models, Greinacher et al. revealed that hexon proteins, components of the adenoviral vectors of the SARS‐CoV‐2 vaccines, can bind to PF4, causing neoantigen presentation. Anti‐PF4 IgG antibodies are produced against these PF4/AV hexon protein complexes which facilitate the activation of platelets in a polyanion‐dependent manner and generate the pro‐coagulant state observed.^23^

As discussed, HIT and VITT antibodies bind to unique sites on PF4 tetramers (Figure 2). The binding of VITT antibodies to the heparin‐binding site on PF4 tetramers was suggested when the VITT antibody epitope was found to consist of eight PF4 amino acids (Arg22, His23, Lys46 and Lys66) consistent with the heparin‐binding site. Interestingly, upon the addition of heparin to VITT samples, there was inhibition of platelet activation, indicating that heparin may displace VITT antibodies from the heparin‐binding site.^19^ This further implies that VITT is mediated via the binding of VITT antibodies to the heparin‐binding site of PF4.^19^, ^23^

Intriguingly, there have been recent reports of a novel anti‐PF4 disorder, which has been termed as a ‘VITT‐like’ anti‐PF4 disorder. This is distinct from HIT and VITT in that, although presenting both clinically and serologically similar to VITT, it cannot be attributed to either exposure to SARS‐CoV‐2 vaccination or heparin but could be related to infective or tissue damage mechanisms. A recent study by Schönborn et al. analysed 188 sera from patients referred with thrombocytopenia and/or thrombosis and strongly positive results in the anti‐PF4/heparin IgG EIA, but negative by heparin‐induced platelet activation (HIPA), obtained prior to the SARS‐CoV‐2 pandemic. They recorded that 13 of these sera samples demonstrated negative (or weak‐positive) heparin‐dependent platelet activation but strong‐positive PF4‐dependent platelet activation (see Laboratory diagnosis of HIT and Diagnosis of VITT and VITT‐like disorders) suggesting patients with results serologically indicative of VITT existed prior to SARS‐CoV‐2 vaccination.^50^

Then, Schönborn et al. identified nine patients, who had not been exposed to either heparin or SARS‐Cov‐2 vaccination, who presented with thrombocytopenia, thrombosis and again, negative HIPA but positive platelet activation in the presence of PF4 (PIPA) platelet activation assays (i.e. HIPA test performed in the presence of PF4 rather than heparin) (see Diagnosis of VITT and VITT‐like disorders). Interestingly, for five of these patients, a history of infection preceded the thrombotic events by 5–14 days, a similar time frame to that seen in VITT patients following adenovirus vector‐based vaccination. This implies that there may be alternative triggers to adenoviral‐based vaccine components and heparin that can drive the development of platelet‐activating IgG antibodies. Moreover, three of the nine patients demonstrated to have VITT‐like antibodies had been previously diagnosed with spontaneous HIT.^50^ Adenovirus infections have been suggested as a possible trigger of VITT‐like disorders and recent observations have described cases of VITT‐like disorders following human papillomavirus (HPV) vaccination or infection with respiratory syncytial virus.^50^, ^51^, ^52^, ^53^

Therefore, further research is warranted into this novel phenomenon of a VITT‐like anti‐PF4 disorder and its distinction from HIT and VITT in their triggering agents and clinical progression to severe thrombotic events. This will allow appropriate diagnosis and treatment of anti‐PF4 disorder patients.

## PATHOGENESIS OF THROMBOSIS AND THROMBOCYTOPENIA IN ANTI‐PF4 DISORDERS

IgG‐mediated cellular activation via Fcγ receptor binding is central to the pathophysiology of anti‐PF4 disorders (Figure 3).^54^ Platelets express only low‐affinity FcγRIIa receptors.^55^ Upon FcγRIIa receptor cross‐linking, mediated by binding to the Fc region of IgG contained in immune complexes (e.g. PF4/H/IgG), there is strong platelet activation through the immunoreceptor tyrosine‐based activation motif (ITAM).^4^, ^56^ Platelets are then transformed into a procoagulant state with increased platelet aggregation and release of platelet‐derived procoagulant microparticles, as well as PF4. Warkentin et al. demonstrated that pathogenic HIT IgG antibodies were able to trigger the platelet procoagulant response, measured in terms of activation status and microparticle release, to the same extent as established platelet agonists such as thrombin and collagen.^7^, ^57^ Huynh et al. showed that, in both HIT and VITT samples, there was complete inhibition of platelet activation by addition of an FcγRIIa‐blocking monoclonal antibody.^19^ Simultaneously, thrombocytopenia is mediated by the clearance of anti‐PF4 immune complex‐bound platelets via macrophages of the reticuloendothelial system.^54^

Anti‐PF4‐IgG immune complexes are also able to activate monocytes and neutrophils, again via Fcγ receptor binding. The role of low‐affinity FcγRIIa receptors versus high‐affinity Fcγ receptors, FcγI and FcγIII, in this process is unclear.^4^, ^56^, ^58^ Purified HIT IgG antibodies have been shown to stimulate monocyte‐mediated tissue factor production, but only when incubated in whole blood with both PF4 and heparin. This demonstrates that, at least for cHIT, immune complex formation, that is, PF4/H/IgG, may be essential for the IgGs to elicit their prothrombotic effects via tissue factor production.^59^ This monocyte activation can also facilitate endothelial cell activation via the binding of HIT antibodies to PF4/glycosaminoglycan complexes at the endothelial cell surface, further promoting a prothrombotic state.^54^

Neutrophil activation and subsequent production of neutrophil extracellular traps (NETs) can lead to the development of thrombosis in HIT. Gollomp et al. used a combination of an endothelialised microfluidic system and a murine passive immunisation model to show that NETs can form complexes with PF4 and HIT antibodies, which provide protection against nuclease digestion^48^ In instances of infection or inflammation, NETs have been shown to contribute to thrombosis via the release of chromatin, which can trap platelets, deactivate natural anticoagulants and bind clotting factors.^60^ Indeed, in HIT mice, Gollomp et al. demonstrated that by inhibiting NET formation through *PADI4* gene disruption or DNase treatment, thrombus propagation was limited, and the severity of thrombocytopenia was decreased. VITT antibodies have also been shown to induce thrombus formation through stimulating neutrophils to release NETs.^61^

## DIAGNOSIS OF HIT, VITT AND VITT‐LIKE SYNDROME

Anti‐PF4 disorders should be considered as ‘clinical–pathological’ conditions requiring both clinical symptom evaluation and laboratory testing to make an informed diagnosis.

### Diagnosis of HIT

#### Evaluating the clinical probability of HIT

According to the British Society for Haematology (BSH) Clinical guidelines for ‘Diagnosis and Management of Heparin‐Induced Thrombocytopenia’, suspected HIT diagnosis is completed via a clinical assessment of disease probability followed by laboratory testing.^62^ The ‘4Ts’ scoring system (Table 2) is the most used clinical assessment^63^ but significant variability has been observed among clinicians when calculating the 4Ts score.

**TABLE 2:** The 4T scoring system of HIT.

4Ts category	Number of points		
	2	1	0
Timing of platelet count fall	Clear onset days 5–10 or platelet fall ≤1 day (prior heparin exposure within 30 days)	Onset after day 10, but not clear	Platelet count falls too early and without recent exposure to heparin
Thrombocytopenia	Platelet count fall >50% and platelet nadir ≥20	Platelet count 30%–50% or platelet nadir 10–19	Platelet count fall <30% or platelet nadir <10
Thrombosis	New thrombosis, skin Necrosis	Progressive or recurrent thrombosis, skin lesions	None
Other causes of thrombocytopenia	No other cause identified	Possible	Definite

*Note*: The 4T scoring system is a clinical diagnostic tool used to evaluate the likelihood of HIT. The scoring system is based on four categories: (1) timing of platelet count fall, (2) degree of thrombocytopenia, (3) presence of thrombosis and (4) exclusion of other causes of thrombocytopenia. Each category is scored from 0 to 2 points based on clinical criteria, with higher scores indicating a greater probability of HIT. Adapted from Reference [65].

#### Laboratory diagnosis of HIT

Laboratory diagnosis of HIT relies on qualitative, quantitative and functional assays (Table 3) to identify clinically significant antibodies that induce pathological platelet activation. It is important to note that no current assay has 100% sensitivity and specificity in the diagnosis of HIT, and therefore, immune and functional assays should be performed in combination if feasible to increase sensitivity and specificity.

**TABLE 3:** Summary of qualitative, quantitative and functional laboratory assays used to diagnose anti‐PF4 disorders, HIT and VITT/VITT‐like syndromes.

	Assay	HIT	VITT/VITT‐like
Qualitative	Lateral flow immunoassay (LFIA)	X	X
	Particle gel immuno and immunofiltration assays (PaGIA/PIFA)	X	
Quantitative	Enzyme‐linked immunosorbent assays (ELISA)	X	X
	Chemiluminescent immunoassays (CLIA)	X	X
	Latex immunoassay (LIA IgGAM)	X	
Functional	Heparin‐induced platelet activation assay (HIPA)	X	
	PF4‐induced platelet activation assay (PIPA)	X	X
	Light transmission aggregometry (LTA)	X	
	Serotonin‐release assay (SRA)	X	X
	Flow cytometry	X	X

*Note*: Various laboratory assays can be used to distinguish between HIT and VITT/VITT‐like cases.^62^, ^74^ ‘X’ indicates that the assay is used to aid laboratory diagnosis of either HIT and/or VITT.

HIT laboratory testing is generally not recommended for low 4Ts scores, unless the score is suspected to be inaccurate or affected by confounding factors such as patients supported by ECMO.^62^, ^64^ For intermediate or high scores, immunoassay testing is recommended, followed by functional assays if the immunoassay is positive and functional testing is available. This approach aligns with the ‘iceberg model’, as only a subset of immunoassay‐positive samples demonstrate positivity in functional assays.^65^Pending confirmation, treatment should be initiated immediately as laboratory results may take hours to days.

Quantitative immunoassays include ELISA, chemiluminescent immunoassays (CLIA) and latex immunoassays (LIA). ELISA methods, especially IgG‐specific assays, are widely used for their high sensitivity (>95%). Strong optical density (OD) values (>1.4) correlate with a higher probability of HIT. IgG‐specific assays provide superior specificity compared to polyspecific methods, improving the predictive value for HIT diagnosis.^66^ CLIA offers a rapid alternative to ELISA, with a turnaround time of approximately 30 minutes. These assays achieve sensitivity >95% and specificity >94%, making them suitable for routine diagnostic laboratories and increase the likelihood of functional assay positivity.^66^, ^67^ LIA detects PF4‐heparin antibodies through competitive inhibition of latex bead agglutination. When combined with a selected cut‐off, it demonstrates high sensitivity and specificity, comparable to functional assays.^68^, ^69^

Functional assays, on the other hand, typically measure platelet activation in patients' serum with heparin.

SRA is considered the gold standard for HIT diagnosis due to its high specificity and sensitivity. It measures serotonin release from activated platelets, but complexity limits its availability to specialised centres.^70^ LTA quantifies platelet aggregation or ATP release in the presence of heparin and patient plasma. Sensitivity ranges from 69% to 85%, with positive results confirmed by inhibition of aggregation using high‐concentration heparin.^71^ Flow cytometry identifies platelet activation markers such as P‐selectin and Annexin V using fluorescent antibodies. It has shown earlier detection capability compared to SRA and reported specificity up to 100% in some studies.^72^, ^73^ Whenever possible, functional assays should be used to confirm the diagnosis of HIT, but for initial screening, IgG‐specific ELISAs or CLIAs are preferred due to their high sensitivity and rapid turnaround.

In HIT‐like disorders that do not present with typical PF4‐heparin IgG antibodies, that is, aHIT and spontaneous HIT, a diagnosis is made by detecting heparin‐independent, but PF4‐dependent, platelet‐activating antibodies.^65^

### Diagnosis of VITT and VITT‐like disorders

Pavord et al. evaluated the United Kingdom's first 220 cases of VITT. They determined the probability of VITT across individual patients according to prespecified criteria (Table 4). However, diagnostic criteria may vary between different reports and geographical location.^33^ These criteria have been used to develop consensus management guidance for clinicians creating treatment plans for individual patients presenting with symptoms suggestive of VITT. Similar to VITT, VITT‐like disorders present with thrombocytopenia, thrombosis frequently in atypical vascular beds and extremely high D‐dimer levels. In most cases, there is a recent infection.

**TABLE 4:** Definition of VITT cases according to criteria produced by an expert haematology panel.

Definition of VITT	Description
Definite VITT	Patent must meet all 5 of the following criteria: Onset of symptoms 5–30 days post SARS‐CoV‐2 vaccination Presence of thrombosis Marked elevation of D‐dimer level (>400 mcg/L FEU) Thrombocytopenia (platelet count <150 × 10^9^/L) Positive ELISA for anti‐PF4 antibodies
Probable VITT	D‐dimer level > 4000 mcg/L FEU but one other criterion not met (i.e. either timing, thrombosis, thrombocytopenia, anti‐PF4 antibodies) or D‐dimer level 2000–4000 mcg/L FEU/unknown with all other criteria present
Possible VITT	D‐dimer level 2000–4000 mcg/L FEU/unknown with one other criterion not met, or two other criteria not met
Unlikely VITT	Platelet count <150 × 10^9^/L, D‐dimer <200 mcg/L without thrombosis, or presence of thrombosis, platelet count >150 × 10^9^/L and D‐dimer <2000 mcg/L FEU, regardless of ELISA result for anti‐PF4 antibodies and/or alternative diagnosis more likely

*Note*: Clinical evaluation of patients to determine if they have ‘Definite VITT’, ‘Probable VITT’, ‘Possible VITT’ or ‘Unlikely VITT’ is dependent on the presence of thrombotic events, D‐dimer levels, degree of thrombocytopenia and anti‐PF4 antibody ELISA results.^33^ Various laboratory assays can be used to distinguish between HIT and VITT/VITT‐like cases.^62^, ^74^

Abbreviation: FEU, fibrinogen‐equivalent unit.

It is important that a diagnosis of VITT can be distinguished from a diagnosis of HIT, as treatment considerations differ somewhat between these two anti‐PF4 disorders.^74^ For example, if using anti‐PF4/heparin enzyme immunoassays, both HIT and VITT antibodies would produce a positive result, despite their structural and functional differences.^25^, ^50^ One method of distinguishing between HIT and VITT/VITT‐like antibodies is by using HIPA and PIPA tests (Table 3). These assays detect platelet activation in response to patient antibodies by incubating patient serum and washed platelets with either heparin (HIPA) or PF4 (PIPA) in a microtitre plate.^24^, ^50^, ^75^

VITT samples will show strong PIPA and will demonstrate a negative (or weak‐positive) result in a HIPA test. While HIT samples will test positive in both HIPA and PIPA assays, consistent with observations that PF4 can enhance the sensitivity of HIT antibodies.^50^ However, it is noted that these HIPA and PIPA tests can only be performed in specialised laboratories and so are limited in their application. Recently, to overcome this, a rapid chemiluminescence assay has been developed which can recognise VITT or VITT‐like antibodies (standard CLIA for HIT does not detect VITT antibodies) but will not detect most HIT antibodies. This assay is a modification of the HemoIL AcuStar HIT‐IgG (PF4‐H), consisting of magnetic particles complexed with PF4 alone, rather than PF4/polyvinyl sulphonate.^74^ This assay has also been applied to the investigation of VITT‐like disorders; in that it can detect the presence of VITT antibodies in patients with no prior SARS‐Cov‐2 vaccination.^50^ Other laboratory tests such as prothrombin time (PT), activated partial thromboplastin time (aPTT), D‐dimer and Clauss fibrinogen are used to assist the diagnosis of VITT.^50^, ^76^

HIPA and PIPA assays can be used to distinguish cHIT from spontaneous HIT and aHIT syndromes as well as VITT/VITT‐like syndromes. However, serological variation between individual aHIT, spontaneous HIT cases and their similarity to VITT/VITT‐like disorders makes distinction between them challenging.^77^ Therefore, clinical evaluation, laboratory testing and possible ‘triggers’ (i.e., infection, surgery or adenovirus vaccination) must be taken into account when diagnosing anti‐PF4 disorders.

## TREATMENT AND MANAGEMENT OF ANTI‐PF4 DISORDERS

### Treatment of HIT

**FIGURE 4:**
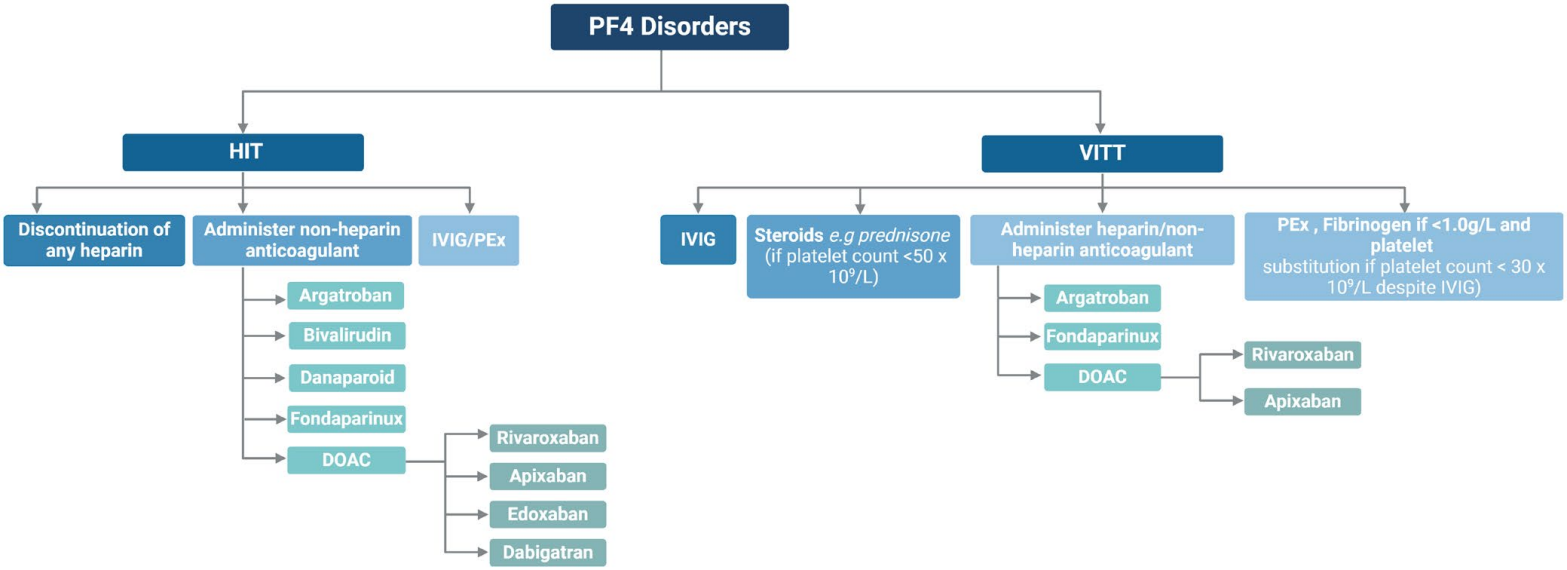
A flow chart illustrating the proposed management and treatment of PF4 disorders HIT and VITT. HIT management involves discontinuation of all forms of heparin and administration of an alternative anticoagulant, with transition to DOACs upon platelet count recovery. VITT management varies depending on the platelet count status and can be treated with heparin or non‐heparin anticoagulant.^50^, ^54^, ^56^, ^57^, ^58^, ^59^, ^60^, ^62^, ^74^ DOAC, direct oral anticoagulant; HIT, heparin‐induced thrombocytopenia; IVIG, intravenous immunoglobulin; PEx, plasma exchange; PF4, platelet factor 4; VITT, vaccine‐induced thrombocytopenia and thrombosis.

The treatment of HIT involves the administration of non‐heparin anticoagulants (generally parenteral in the acute setting) upon clinical suspicion of the diagnosis, and avoidance of any form of heparin, including heparin‐containing flushes and catheters (Figure 4). The disorder is then self‐limiting.

BSH guidelines recommend at least 3 months of anticoagulation in patients with acute HIT complicated by thrombosis (HITT) and 4 weeks of for those without thrombosis.^62^ The choice of anticoagulant may depend on drug factors (e.g. availability, cost, monitoring requirements, administration route, half‐life), patient factors (e.g. kidney and liver function, bleeding risk, clinical stability) and the clinician's expertise.

#### Parenteral anticoagulation therapy

The most commonly used alternative anticoagulant is argatroban, a direct thrombin inhibitor (DTI), administered via continuous IV infusion. Argatroban can be monitored with aPTT, but this is confounded by other factors including the APTT method and reagents as well as the presence of coagulopathies or lupus anticoagulant.^62^ Hence, more specific assays such as the dilute thrombin time (dTT), ecarin clotting time (ECT) or ecarin chromogenic assay (ECA) are preferred. French Guidelines proposed a therapeutic window of 0.5–1.5 μg/mL while the Swiss Society of Hematology Working Party of Hemostasis recommends 0.4–1.5 μg/mL using a calibrated dTT.^78^, ^79^ However, the assay is not available in many laboratories.^62^ Modification of initial doses of argatroban is recommended by the American College of Chest Physicians for patients suffering from heart failure, liver dysfunction, anasarca and recent cardiac surgery.^80^

Bivalirudin is considered a safe and effective treatment for suspected and confirmed HIT, with a low thrombosis rate and no major haemorrhages. However, dose reduction is needed in moderate to severe renal dysfunction. While Bivalirudin is monitored commonly using aPTT,^62^ a direct thrombin inhibitor (DTI) assay can be used to monitor drug activity.^81^

Danaparoid sodium and fondaparinux are additional options for parenteral anticoagulation in HIT. Danaparoid can be administered either via IV infusion or subcutaneous injection, while fondaparinux is given subcutaneously. As fondaparinux‐induced HIT is rare, fondaparinux's use remains feasible in these cases. Both agents are primarily cleared through renal excretion, and their plasma levels can be monitored using calibrated anti‐Xa assays when necessary.^82^

Platelet counts in these patients recover within 7 days in 90% of cases.^83^ In a small subset of patients, however, it may take weeks for complete recovery.^83^ Parenteral anticoagulants are highly effective in preventing future thrombotic events when initiated promptly; however, treatment should continue for at least 48 h and until the platelet count has recovered, as the highest risk period for thrombosis is following heparin cessation. HIT antibodies are transient and generally disappear with a median of 50–85 days, depending on the assay.^84^ In a study of 144 patients with positive HIT antibodies, the median time to a negative test according to the Kaplan–Meier analysis was 50 days (95% CI, 32–64) based on SRA and 85 days (95% CI, 64–124) in the case of the antigen assay.^84^

Patients who developed HIT should ideally avoid exposure to heparin unless it is essential in specific circumstances such as cardiac surgery. Screening for platelet‐activating PF4–heparin antibodies is recommended in such cases.^62^ If the platelet‐activating PF4–heparin antibodies are positive and surgery cannot be delayed, heparin should be avoided, and an alternative anticoagulant such as argatroban or bivalirudin should be used. Patients with a history of HIT who are antibody negative can receive intraoperative unfractionated heparin (UFH) in preference to other anticoagulants, which are less validated for this purpose. However, preoperative and postoperative anticoagulation should be with an anticoagulant other than UFH or LMWH.^62^

#### Oral anticoagulation therapy

Vitamin K antagonists, such as warfarin, should only be initiated after the platelet count has normalised following a HIT diagnosis and with concurrent parenteral therapy until the INR reaches therapeutic levels for >24 h.^62^, ^85^ Direct thrombin inhibitors, such as argatroban and bivalirudin, can affect INR measurements. Therefore, it is crucial to set a target INR of 4 and to reassess the INR 4–6 h after discontinuing these inhibitors to ensure an accurate reading. Direct‐acting oral anticoagulants (DOACs) can serve as alternative anticoagulants in patients with HIT with or without thrombosis^83^, ^86^ usually once the patient is stable and the platelet count is improving.^62^, ^87^ DOACs are not recommended in HIT patients with arterial thrombosis.^88^

#### IV immunoglobulins and/or plasma exchange

Because this is an antibody‐mediated disorder, treatment with IV immunoglobulins (IVIG) and/or plasma exchange (PEx) may be considered in patients with severe HIT syndrome requiring urgent surgery, patients with aHIT, delayed HIT, unavailability of non‐heparin anticoagulants or in patients with severe bleeding where therapeutic anticoagulation cannot be used.^62^ IVIG facilitates the clearance of anti‐PF4 antibodies, as well as inhibiting activation of platelets through inhibition of their FcγRIIa receptors.^89^

### Treatment of VITT/VITT‐like syndrome

As per the International Society on Thrombosis and Haemostasis (ISTH) guidance, patients with VITT should receive IVIg and, if platelet counts fall below 50 × 10^9^/L, steroids such as prednisone should be considered.^74^, ^76^ Bleeding risk should be assessed prior to the administration of anticoagulants such as fondaparinux, argatroban or direct oral anticoagulants such as rivaroxaban or apixaban. Although it was thought initially that heparin should be avoided in patients with VITT or VITT‐like syndrome, there are reported cases of successful use of heparin in patients with VITT, and based on the pathophysiology, it is unlikely that heparin would increase the risk of thrombosis. Fibrinogen substitution and PEx are considered if platelet counts remain at less than 30 × 10^9^/L and patients are at a high risk of bleeding despite IVIg.^76^, ^90^ Treatment of VITT or VITT‐like syndrome is summarised in Figure 4.

At present, there is exceptionally little research into the pathogenesis of VITT‐like syndromes and their clinical/serological characterisation. With this, it is noted that there are currently no distinct treatments between VITT and VITT‐like syndromes.

## CONCLUSIONS AND FUTURE DIRECTIONS

The recognition of VITT and the more recent recognition of a VITT‐like anti‐PF4 disorder has and will continue to advance understanding of anti‐PF4 disorders. Further understanding of HIT pathophysiology has highlighted the potential for targeted, site‐specific interventions such as disrupting antigen formation and protecting the endothelium, which could mitigate thrombotic complications while preserving systemic haemostasis. With VITT cases becoming less common due to the discontinuation of both Oxford‐AstraZeneca and Johnson and Johnson/Janssen SARS‐CoV‐2 vaccines, future research should focus on VITT‐like disorders in order to elucidate alternative triggers of anti‐PF4 antibodies and to continue to develop the diagnostic criteria and treatment options.

## AUTHOR CONTRIBUTIONS

MVP and DVP contributed to literature search and drafted the manuscript. ML wrote and revised the manuscript. DJA conceptualised and designed the manuscript, wrote and revised the manuscript. All authors reviewed and approved the final version of the manuscript.

## FUNDING INFORMATION

DJA is funded by Medical Research Council UK (MR/Z505274/1) and infrastructure support for some of the work and funding for MVP were provided by the NIHR Imperial Biomedical Research Centre (BRC).

## CONFLICT OF INTEREST STATEMENT

The authors declare no competing interests.
